# Changes in Blood Markers of Oxidative Stress, Inflammation and Cardiometabolic Patients with COPD after Eccentric and Concentric Cycling Training

**DOI:** 10.3390/nu15040908

**Published:** 2023-02-11

**Authors:** Mayalen Valero-Breton, Denisse Valladares-Ide, Cristian Álvarez, Reyna S. Peñailillo, Luis Peñailillo

**Affiliations:** 1 Exercise and Rehabilitation Sciences Institute, School of Physical Therapy, Faculty of Rehabilitation Sciences, Universidad Andres Bello, Santiago 7550196, Chile; 2 Long Active Life Laboratory, Instituto de Ciencias de la Salud, Universidad de O’Higgins, Rancagua 2841959, Chile; 3 Laboratory of Reproductive Biology, Center for Biomedical Research and Innovation (CIIB), Universidad de los Andes, Santiago 7620001, Chile

**Keywords:** pulmonary rehabilitation, chronic obstructive pulmonary disease, aerobic training, exercise prescription

## Abstract

Chronic obstructive pulmonary disease (COPD) patients manifest muscle dysfunction and impaired muscle oxidative capacity, which result in reduced exercise capacity and poor health status. This study examined the effects of 12-week eccentric (ECC) and concentric (CONC) cycling training on plasma markers of cardiometabolic health, oxidative stress, and inflammation in COPD patients. A randomized trial in which moderate COPD was allocated to ECC (*n* = 10; 68.2 ± 10.0 year) or CONC (*n* = 10; 71.1 ± 10.3 year) training groups. Participants performed 12-week ECC or CONC training, 2–3 sessions per week, 10 to 30 min per session. Before and after training, peak oxygen consumption, maximal power output (VO_2peak_ and PO_max_), and time-to-exhaustion (TTE) tests were performed. Plasma antioxidant and oxidative markers, insulin resistance, lipid profile, and systemic inflammation markers were measured before and after training at rest. VO_2peak_, PO_max_ and TTE remained unchanged after ECC and CONC. CONC induced an increase in antioxidants (*p* = 0.01), while ECC decreased antioxidant (*p* = 0.02) markers measured at rest. CONC induced lesser increase in oxidative stress following TTE (*p* = 0.04), and a decrease in insulin resistance (*p* = 0.0006) compared to baseline. These results suggest that CONC training induced an increase in insulin sensitivity, antioxidant capacity at rest, and lesser exercise-induced oxidative stress in patients with moderate COPD.

## 1. Introduction

Chronic obstructive pulmonary disease (COPD) is characterized by airflow limitation associated with inflammation of the airway [1]. A common extra-pulmonary manifestation of patients with COPD is skeletal muscle dysfunction, which is evidenced mainly by reduced muscle endurance and strength of the lower limbs [2]. Impaired muscle oxidative capacity, a shift toward a glycolytic muscle fiber type distribution, reduced capillarity, and reduced cross-sectional area of muscle fiber led to muscle dysfunction in COPD patients [2]. The reduced exercise capacity and increased fatigability are clinical manifestations that exacerbate poor health status and limit daily life activities in COPD patients [3]. The limited exercise capacity of these individuals also predisposes them to develop impaired metabolism of glucose and lipids, which may lead to metabolic syndrome [4]. Airway inflammation releases inflammatory molecules into the bloodstream, thus, COPD patients show evidence of an increased level of systemic inflammation chronically [2]. It has been shown that inflammation can increase muscle degradation, inhibiting muscle-specific protein expression, and increasing muscle cell apoptosis, worsening muscle dysfunction in COPD patients [5]. Thus, systemic inflammation has been proposed as a mechanism in the development of muscle dysfunction in COPD patients as interleukin-6 (IL-6), interleukin-1 beta (IL-1β), and tumor necrosis factor-alpha (TNF-α) have been shown to be elevated in these patients [6]. Based on the evidence, it is accepted that COPD patients also have elevated levels of oxidative stress due to the imbalance between the generation of reactive oxygen species (ROS) and the efficiency of the antioxidant mechanism [7]. All these manifestations affect skeletal muscle function and further worsen muscle dysfunction. Thus, searching for new rehabilitation strategies to reduce muscle dysfunction within the burden of this disease is necessary.

Several studies have shown that exercise training is the cornerstone of pulmonary rehabilitation as it markedly improves functional performance, muscle mass, and exercise capacity in COPD patients [8,9,10]. Endurance exercise training is the most common exercise modality prescribed in COPD [11,12,13], which has been shown to improve exercise tolerance and muscle oxidative capacity in these patients [12,14,15,16]. However, endurance training could also increase oxidative stress levels in severe COPD patients [17], which may not be desirable as it could impair skeletal muscle function. In contrast, another novel and less explored exercise training modality is eccentric cycling training. This type of training is characterized by eccentric contractions of the lower limb muscles that are performed when resisting the backward rotational movement of the cranks generated by an eccentric ergometer. Notably, eccentric cycling imposes lesser cardiopulmonary, metabolically, and perceptual demands (i.e., lower oxygen consumption, dyspnea, and blood pressure) than concentric cycling, making it safer for COPD patients [18]. Interestingly, eccentric cycling can produce a greater workload for the same metabolic demand than concentric cycling in COPD patients [18,19]. Furthermore, eccentric cycling training has been shown to increase muscle strength and mass to a greater extent than concentric cycling in moderate COPD patients [19,20], and a decrease (20%) in the homeostasis model assessment of insulin resistance index (HOMA-IR) after 12 weeks of continuous moderate-intensity eccentric cycling training [21]. Notably, these benefits were reported despite ~30-50% lesser cardiovascular strain and metabolic cost than conventional concentric cycling [21]. Interestingly, we have recently shown that oxidative stress levels (i.e., thiobarbituric acid reactive substances; TBARS) decreased after one acute bout of eccentric cycling at moderate intensity [22]. Hence, eccentric cycling training could be an ideal exercise modality for COPD patients. However, the long-term effects of eccentric cycling training on oxidative stress and inflammation in COPD patients have not been explored yet.

Interestingly, exercise training in COPD patients has been shown to produce positive [23], null [24], or negative changes in oxidative stress assessed at rest [25]. Discrepancies among studies may be due to differences in exercise prescription and heterogenicity of patients’ severity. However, it is possible that in rest conditions, the oxidative molecules are not produced/released, and the antioxidant capacity is not fully displayed, hence, no changes have been observed. A single bout of exercise has been shown to induce exaggerated production of ROS and oxidative damage in COPD patients when exercise exceeds a certain intensity or duration [26]. Thus, it seems plausible to assess the oxidative and antioxidant response to an acute bout of exercise to dynamically assess oxidative stress handling in COPD patients. However, this has not been explored yet. There are tests that are used to evaluate exercise tolerance, such as time-to-exhaustion tests (TTE) [27]. Specifically, TTE consists of the patient sustaining a fixed workload for the longest time possible, which has been used in COPD patients [28]. However, to the best of our knowledge, no study has examined oxidative stress and antioxidant markers after TTE in COPD patients after an intervention of exercise training. Therefore, as oxidative stress has been involved in muscle mass loss and dysfunction in COPD patients [29,30], assessing oxidative stress changes after TTE in COPD patients could shed light on exercise-induced oxidative stress handling in these patients.

Thus, this study aimed to examine the effects of 12 weeks of eccentric (ECC) and conventional concentric (CONC) cycling training on markers of oxidative stress, inflammation, and cardiometabolic health in COPD patients measured at rest. Furthermore, as oxidative stress could be induced by acute exercise, we compared the changes in oxidative stress markers following a standardized submaximal exercise between ECC and CONC, before and after training in COPD patients. 

## 2. Materials and Methods

### 2.1. Participants

This study is part of a larger study, further details of participants and training interventions have been published elsewhere [19]. In brief, it was a randomized prospective training study, in which twenty participants with moderate COPD (10 men and 10 women) volunteered to participate. Participants were diagnosed as moderate COPD patients according to the Global Initiative for Chronic Obstructive Lung Disease (GOLD; II) based on a pulmonologist assessment and spirometry tests. The sample size was estimated based on a reduction of 8.8% in total cholesterol levels after eccentric training (effect size of 1.5) reported in a previous study [31]. Considering an alpha level of 0.05 and a statistical power of 0.8, the sample size estimation revealed that eight participants per group would be sufficient (G * Power 3.1, Germany). Ten participants per group were included to account for a 20% dropout.

The exclusion criteria considered supplemental oxygen therapy, kidney, neurologic or cardiovascular disease, recent exacerbation with three or more days of steroid or antibiotic use, being part of strength or aerobic training in the last year; musculoskeletal injury of the legs during the past one year, and smoking in the last six months. Participants were instructed to refrain from other types of training and from any nutritional supplementation and to maintain regular daily routines during the study period. Written informed consent was obtained from all participants. The study was approved by the Institutional Ethics Committee and Eastern Metropolitan Health Service of the city (clinical trial registration number: DRKS00009755) and conducted according to the Helsinki declaration. 

### 2.2. Study Design

Participants were allocated to ECC (men: 6, women: 4) or CONC (men: 4, women: 6), using a stratified randomized allocation scheme considering FEV_1_ and age to be similar between groups. The physical characteristics of the participants are presented in the Results. All testing was completed in the laboratory at room temperature (20–22 °C) at the same time of day (±1 h). Participants were asked not to consume caffeine and alcohol for at least 3 h and 24 h, respectively.

Outcome measurements were collected 72 h before and after 12 weeks of ECC and CONC training. Participants were cited to the laboratory on two different days to complete all assessments. On day one, an incremental cycling test was performed to determine peak oxygen consumption (VO_2peak_) and maximal concentric power output (PO_max_). Forty-eight hours after, participants returned to the laboratory, and blood was withdrawn from the antecubital vein was collected at rest after 12 h of fasting. Furthermore, another blood sample was collected following a time-to-exhaustion cycling test (TTE). Participants received a light breakfast consisting of a cereal bar (78.4 kCal of energy; 1.1 g protein, 2.5 total fat, 13 g carbohydrate) and a milk box (40 kCal of energy; 0 g protein, 0 total fat, 8 g carbohydrate) after the first blood withdraw at rest. Immediately following the TTE a second blood sample was collected to determine the changes (Δ: Post-TTE—Pre-TTE) in plasma oxidative stress markers to determine exercise-induced oxidative stress.

### 2.3. Exercise Training

Participants attended four sessions before the commencement of the training to familiarize patients with exercise. During familiarization sessions, eccentric or concentric cycling was performed (5–20 min) at ~25% of their self-perceived maximum effort. After this, participants performed eccentric or concentric cycling training for 12 weeks with a total number of training sessions of 34. Training frequency started with two times per week, on weeks 1–2, and progressed to three times per week from weeks 3 to 12. Training intensity was moderate and increased from 11 to 13 on the rating of perceived exertion (RPE) from a 6-20 Borg’s scale. Training time started from 10 min in week 1 to 30 min in week 12. Training total workload was different between CONC and ECC as for the same RPE, the ECC training group performed a much higher training average workload ECC = 226.6 ± 101.9 vs. CONC = 78.1 ± 62.7 kJ; *p* ≤ 0.05). The CONC group trained in a conventional concentric recumbent cycle ergometer (Livestrong, LS 5.0R model, Austin, TX, USA). The ECC group trained in a recumbent eccentric cycle ergometer (Eccentric Trainer, Metitur, Finland) in which participants were instructed to resist the backward movements of the cranks, which is known to induce eccentric contractions of the knee extensor muscles mainly. All training sessions were performed in an airconditioned laboratory (temperature: 20–21 °C, relative humidity: 50%). Exercise training protocol induced changes in body composition and functional performance markers after training, which have been published previously elsewhere [19].

### 2.4. Measurements

#### 2.4.1. Maximal Incremental Cycling Test

On day one, patients performed a maximal incremental test on an electromagnetically braked ergometer (Livestrong, LS 5.0 R model, Austin, TX, USA). The test started at 10 W for two minutes, with 10 W increase every minute until voluntary exhaustion or until the patients were unable to maintain 60 revolutions per minute of cadence [18]. Oxygen consumption (VO_2_) and carbon dioxide production (VCO_2_) were assessed using a breath-by-breath gas analyzer (Ergocard, Medisoft, Belgium). PO_max_ and VO_2peak_ were obtained as described in previous study [32].

#### 2.4.2. Time-to-Exhaustion Test (TTE)

On day two, a constant power output time-to-exhaustion cycling test was performed at 75% of PO_max_ [33]. Participants were instructed to adopt their preferred cadence between 60 and 90 rpm and maintain the target power output for as long as possible. Verbal encouragement was provided; however, participants were not given feedback on their elapsed time or power output. The participants’ TTE was reached when despite encouragement, their cadence fell 10 rpm below 60 rpm for 10 s or more. The TTE was recorded to the nearest second. Total work was calculated by multiplying time by average power output and expressed in kJ [27].

#### 2.4.3. Blood Samples

As mentioned above, an initial blood sample was obtained from the antecubital vein following a 12 h overnight fast at rest before and 72 h after the last training session. Additionally, a second blood sample was collected following (~5 min) the TTE. Blood samples were collected in Vacutainer© EDTA tubes for plasma and tubes with pro-coagulant (10 mL) for serum. Tubes were centrifuged for 10 min at 4 °C and 4000 rpm. Plasma and serum were aliquoted in Eppendorf tubes with 500 μL of sample and stored at −80 °C until analyzed.

#### 2.4.4. Oxidative Stress and Inflammatory Markers

Oxidative stress and inflammatory markers were analyzed by ELISA or colorimetric kits (Cayman Chemical Company, Ann Arbor, MI, USA) and read on a plate reader (Multiskan FC, Thermo Scientific, Beijing, China). All measurements were performed in plasma or serum samples according to the manufacturer’s instructions. Interleukin 6 (IL-6), tumor necrosis factor α (TNF-α), interleukin 1 beta (IL-1β), total antioxidant capacity (TAC), superoxide dismutase (SOD), catalase (CAT), thiobarbituric acid reactive substances (TBARS) and glutathione peroxidase activity (GPx) were analyzed at rest (pre-TTE). Changes (Δ: Post-Pre) in these markers after TTE were also determined (TACTTE, SODTTE, CATTTE, TBARSTTE, GPxTTE). All samples were analyzed in duplicate. Coefficient of variation of duplicates were: IL-6: 5.2 ± 5.0%, TNF-α: 21.5 ± 18.4%, IL-1β: 21.9 ± 26.8%, TAC: 9.8 ± 7.2%, SOD: 5.6 ± 7.9%, CAT: 4.4 ± 3.6%, TBARS: 3.1 ± 3.7%, and GPx: 4.4 ± 3.4%.

#### 2.4.5. Cardiometabolic Health Markers

##### Insulin Sensitivity

Fasting plasma glucose (FPG) concentration was measured using an enzymatic method (Trinder, Genzyme Diagnostics, Charlottetown, Canada) and plasma insulin was assessed using a radioimmune assay (DPC, Houston, TX, USA). Homeostatic model assessment insulin resistance (HOMA-IR) was calculated according to Matthew’s equation [34]. Whole-blood glycosylated hemoglobin (%Hb1ac) was measured using a latex immunoturbidimetric assay in a DCA analyzer (Siemens Medical, Devault, PA, USA).

##### Lipid Profile

Total cholesterol (TC) and triglycerides (TAG) concentrations were analyzed by enzymatic methods using standard kits (Wiener Lab Inc., Rosario, Argentina) in an automatic analyzer (Metrolab 2 300 Plus™, Metrolab Biomed Inc., Rosario, Argentina). Plasma high-density lipoprotein cholesterol (HDL) levels were measured by the same enzymatic method after phosphotungstate precipitation. Low-density lipoprotein cholesterol (LDL) levels were calculated using the Friedewald formula.

### 2.5. Statistical Analysis

The Shapiro–Wilk test was used to assess the distribution of the data as a whole deviates from a comparable normal distribution, and the analysis showed that all dependent variables were normally distributed. Baseline characteristics between groups were analyzed by Student *t* test. Changes in the dependent variables from the baseline to post-training were compared between the ECC and CONC groups by a two-way repeated measure analysis of variance. If a significant interaction (group × time) effect, was found a Fisher’s least significance difference (LSD) post hoc test was used. The Mann–Whitney test was used to compare the percentage of change in oxidative stress variables and cardiometabolic health markers. Statistical significance was set at *p* ≤ 0.05, and data are presented as mean and standard deviation (mean ± SD). All statistical analyses were performed using PRISM 8.0 (GraphPad, San Diego, CA, USA).

## 3. Results

### 3.1. Participants’ Characteristics

Age (ECC = 68.2 ± 10.0 year, CONC = 71.1 ± 10.3 year; *p* = 0.53), body mass (ECC = 73.2 ± 11.7 kg, CONC = 69.7 ± 10.0 kg; *p* = 0.47), height (ECC = 161.2 ± 7.4 cm, CONC = 158.5 ± 12.0 cm; *p* = 0.54), body mass index (ECC = 29.1 ± 5.4 kg/m^2^, CONC = 28.1 ± 5.9 kg/m^2^; *p* = 0.96), forced expiratory volume in 1 second to forced vital capacity ratio (ECC = 63.1 ± 0.9, CONC = 66.8 ± 1.2; *p* = 0.42), and forced expiratory volume in 1-second post-bronchodilator (ECC = 68.7 ± 15.1% of predicted, CONC = 73.1 ± 12.8% of predicted; *p* = 0.49) were similar between groups.

### 3.2. Maximal Aerobic Capacity

**Figure 1:**
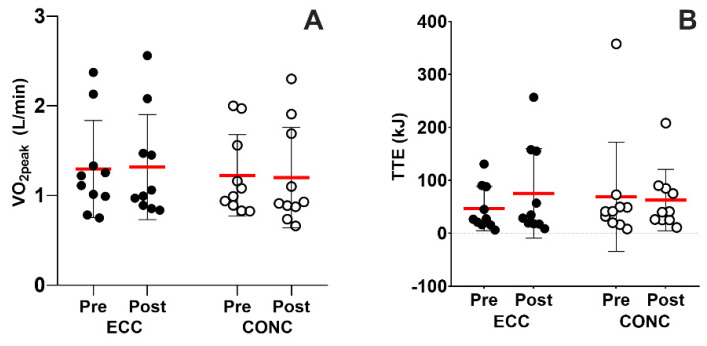
Maximal and submaximal aerobic performance. Peak oxygen consumption during the incremental cycling test (**A**) and workload performed in the time to exhaustion test (**B**). ECC: eccentric cycling, CONC: concentric cycling.

The $\dot{V}$O_2peak_ (1.2 ± 0.5 L/min and 1.2 ± 0.4 L/min; *p* = 0.8) and PO_max_ (87.0 ± 54.1 W and 76.0 ± 37.1 W; *p* = 0.6) were similar at baseline between ECC and CONC, respectively. Moreover, as shown in Figure 1A, $\dot{V}$O_2peak_ of both groups remained unchanged (*p* > 0.05) after training (ECC, Post: 1.3 ± 0.6, CONC, Post: 1.2 ± 0.6 L/min). PO_max_ also remained unchanged after ECC (92.0 ± 55.5 W) and CONC (98.0 ± 54.3 W) training (*p* > 0.05).

### 3.3. TTE

As shown in Figure 1B, total work performed in TTE in ECC (46.4 ± 41.7 kJ) and CONC (68.6 ± 103.3 kJ) were similar at baseline (*p* = 0.2). Moreover, both groups remained unchanged (*p* > 0.05) after training (ECC, Post: 75.1 ± 84.6, CONC, Post: 62.4 ± 58.1 kJ).

### 3.4. Systemic Oxidative Stress Markers

#### 3.4.1. At Rest

**Figure 2:**
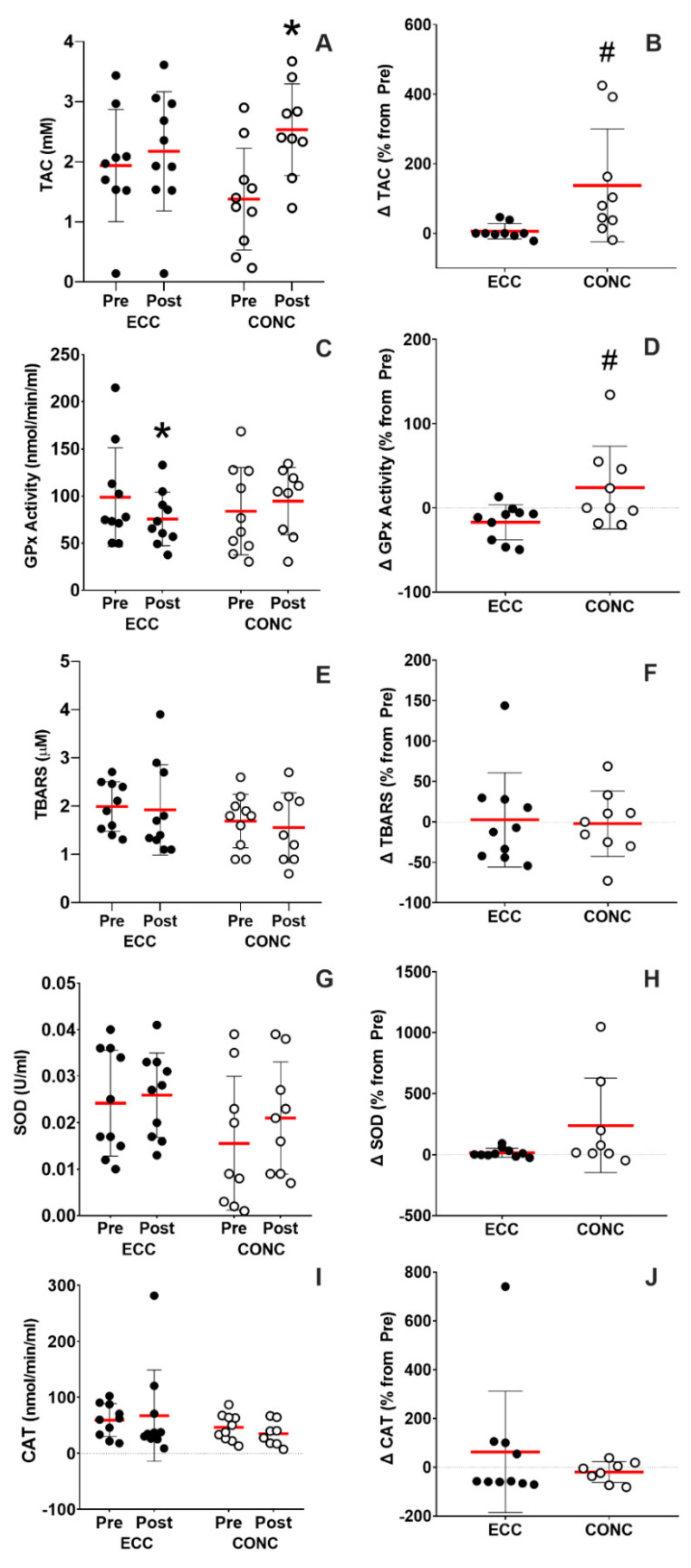
Oxidative stress at rest. Total antioxidant capacity (TAC) levels in absolute values (**A**) as percentage of change from pre-training values (**B**). Glutathione peroxidase activity (GPx) in absolute values (**C**) as percentage of change from pre-training values (**D**). Thiobarbituric acid reactive substances (TBARS) levels in absolute values (**E**) as percentage of change from pre-training values (**F**). Superoxide dismutase (SOD) levels in absolute values (**G**) as percentage of change from pre-training values (**H**). Catalase (CAT) levels in absolute values (**I**) as percentage of change from pre-training values (**J**). ECC: eccentric cycling, CONC: concentric cycling. *: *p* < 0.05 vs. Pre-. #: *p* < 0.05 ECC vs. CONC.

As shown in Figure 2A, TAC concentration at rest showed a 137.9 ± 162.4% increase after CONC training (time effect, *p* = 0.004; training effect, *p* = 0.6; interaction effect, *p* = 0.02). Moreover, GPx activity decreased by 17.2 ± 20.8% after ECC training (time effect, *p* = 0.3; training effect, *p* = 0.9; interaction effect, *p* = 0.03) as shown in Figure 2C. In addition, the percentages of changes from baseline in both oxidative stress markers were statistically different as shown in Figure 2B (TAC, *p* = 0.01) and Figure 2D (Gpx activity, *p* = 0.02). Other oxidative stress markers measured at rest (Figure 2E–J) remained unchanged after ECC and CONC training: TBARS (time effect, *p* = 0.6; training effect, *p* = 0.1; interaction effect, *p* = 0.8), SOD (time effect, *p* = 0.1; training effect, *p* = 0.2; interaction effect, *p* = 0.5) and CAT (time effect, *p* = 0.9; training effect, *p* = 0.1; interaction effect, *p* = 0.5).

#### 3.4.2. Submaximal Cycling

**Figure 3:**
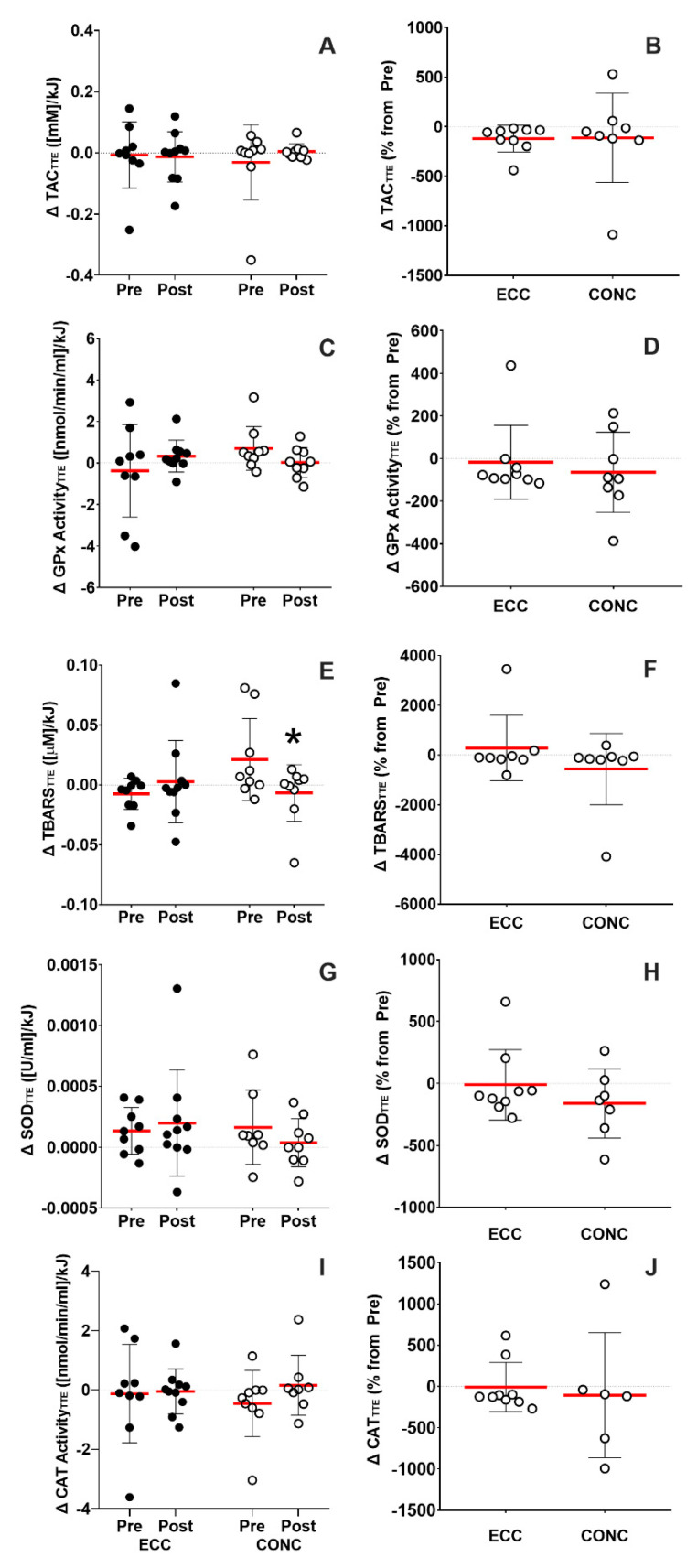
Oxidative stress markers change from rest to immediately following time to exhaustion test (TTE) normalized by the total workload during TTE. Absolute value changes in total antioxidant capacity (TAC_TTE_) (**A**) and as a percentage of change from baseline (**B**). Absolute value changes in glutathione peroxidase activity (GPx_TTE_) (**C**) and as a percentage of change from baseline (**D**). Absolute value changes in thiobarbituric acid reactive substances (TBARS_TTE_) (**E**) and as a percentage of change from baseline (**F**). Absolute value changes in superoxide dismutase (SOD_TTE_) (**G**) and as a percentage of change from baseline (**H**). Absolute value changes in catalase (CAT_TTE_) (**I**) and as a percentage of change from baseline (**J**). ECC: eccentric cycling, CONC: concentric cycling. *: *p* < 0.05 vs. Pre-.

Changes in oxidative markers following TTE are shown in Figure 3. Oxidative stress values obtained were normalized by workload performance on the TTE of each patient in kJ. Absolute value changes in TAC_TTE_ were similar after ECC and CONC training (time effect, *p* = 0.5; training effect, *p* = 0.9; interaction effect, *p* = 0.4; Figure 3A) and no differences were found in the percentages of change from baseline in any group (*p* = 0.5; Figure 3B). Changes in absolute values of GPx_TTE_ were similar after ECC and CONC (time effect, *p* = 0.5; training effect, *p* = 0.9; interaction effect, *p* = 0.4; Figure 3C), similar to when the percentage of change from baseline were compared (*p* = 0.6; Figure 3D). The absolute values of TBARS_TTE_ decreased 244.8 ± 566.5% only after CONC training (*p* = 0.04; Figure 3E), but no difference was found in the percentage of change from baseline between groups in this marker (*p* = 0.5; Figure 3F). No significant changes in absolute values were found in SOD_TTE_ (time effect, *p* = 0.7; training effect, *p* = 0.5; interaction effect, *p* = 0.3; Figure 3G) and CAT_TTE_ (time effect, *p* = 0.3; training effect, *p* = 0.8; interaction effect, *p* = 0.4; Figure 3I) after any training. Furthermore, percentages of change from baseline of SOD_TTE_ (*p* = 0.4) and CAT_TTE_ (*p* = 0.4) were similar between ECC and CONC (Figure 3).

### 3.5. Systemic Inflammation Markers at Rest

**Figure 4:**
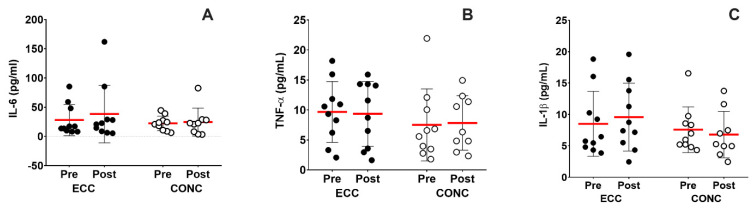
Concentration of systemic inflammation markers. Interleukin 6 (IL-6) (**A**), tumor necrosis factor α (TNF-α) (**B**), and interleukin 1 (IL-1) (**C**) concentrations. ECC: eccentric cycling, CONC: concentric cycling.

The IL-6 (time effect, *p* = 0.2; training effect, *p* = 0.4; interaction effect, *p* = 0.6; Figure 4A), TNF-α (time effect, *p* = 0.9; training effect, *p* = 0.3; interaction effect, *p* = 0.8; Figure 4B) and IL-1β (time effect, *p* = 0.5; training effect, *p* = 0.4; interaction effect, *p* = 0.5; Figure 4C) concentrations remained unchanged after ECC and CONC training.

### 3.6. Cardiometabolic Health Markers

#### 3.6.1. Insulin Sensitivity

**Figure 5:**
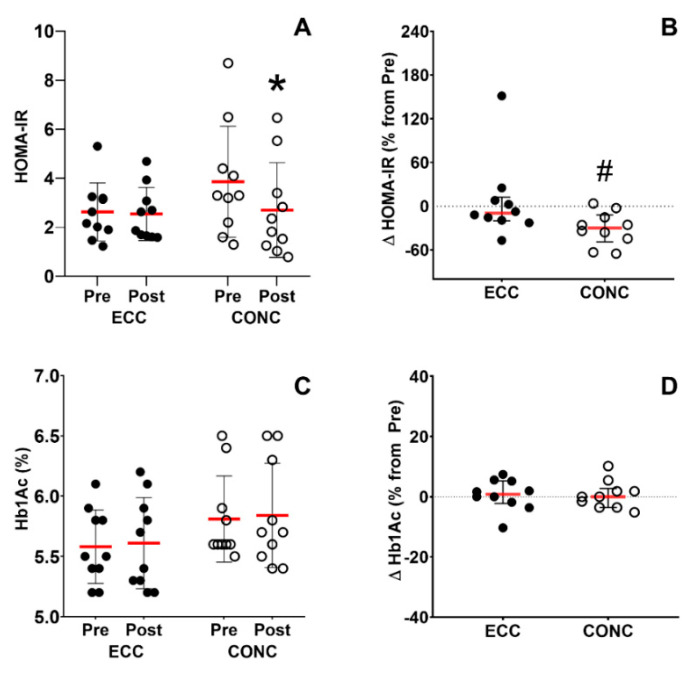
Insulin sensitivity. Homeostatic model assessment insulin resistance (HOMA-IR) in absolute values (**A**) and as a percentage of change from pre-training values (**B**). Whole-blood glycosylated hemoglobin (%Hb1ac) in absolute values (**C**) and as a percentage of change from pre-training values (**D**). ECC: eccentric cycling, CONC: concentric cycling. *: *p* < 0.05 Pre vs. Post, #: *p* < 0.05 ECC vs. CONC.

The HOMA-IR index decreased (−30.8 ± 22.9%) after CONC (from 3.86 ± 2.27 to 2.7 ± 1.93), but not after ECC training (time effect, *p* = 0.005; training effect, *p* = 0.3; interaction effect, *p* = 0.01; Figure 5A). The percentage of change in HOMA-IR showed a greater decrease in CONC compared to ECC (*p* = 0.03; Figure 5B). No significant changes in %Hb1Ac were observed after ECC or CONC training (time effect, *p* = 0.6; training effect, *p* = 0.1; interaction effect, *p* > 0.9; Figure 5C). No differences in the percentage of change after training in %Hb1Ac were observed after ECC or CONC (*p* = 0.7; Figure 5D).

#### 3.6.2. Lipid Profile

**Figure 6:**
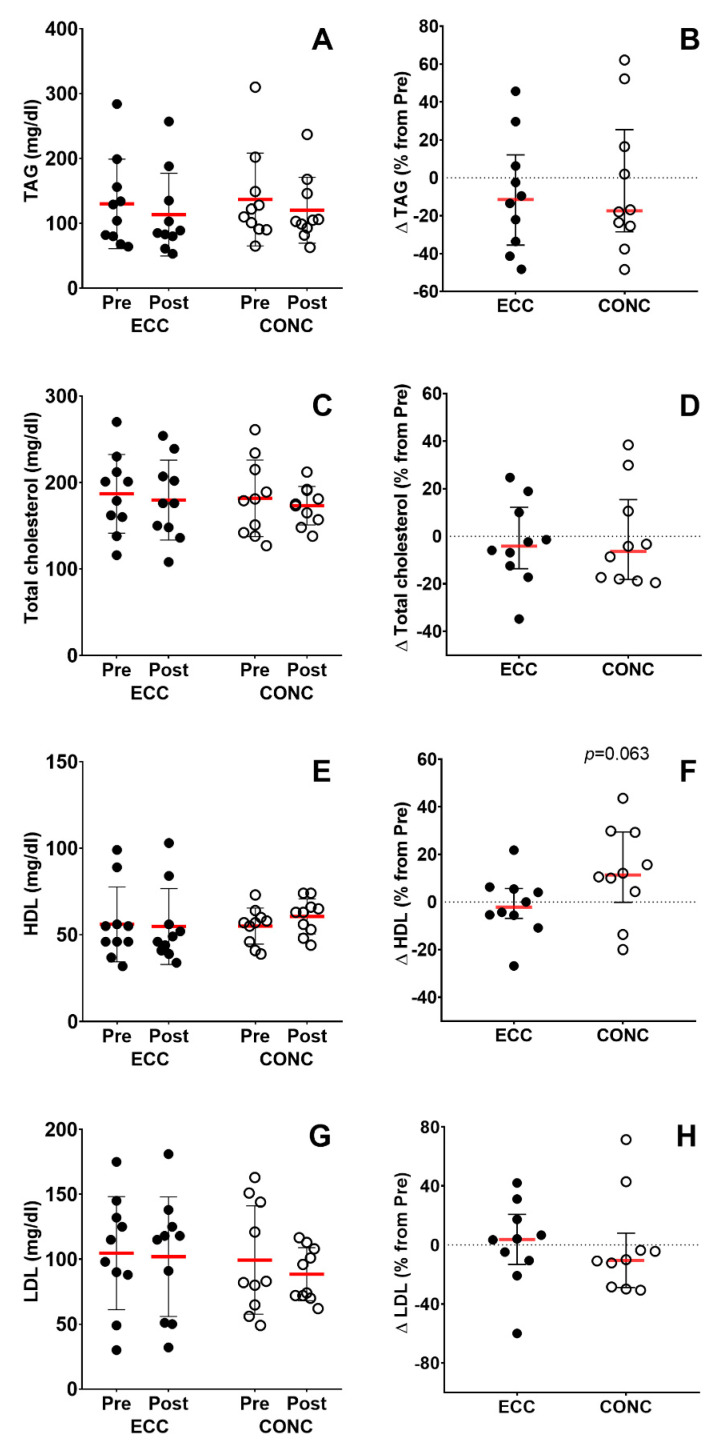
Lipid profile. Triglycerides (TAG) levels in absolute values (**A**) as a percentage of change from pre-training values (**B**). Total cholesterol levels in absolute values (**C**) as a percentage of change from pre-training values (**D**). High-density lipoprotein cholesterol (HDL) levels in absolute values (**E**) as a percentage of change from pre-training values (**F**). Low-density lipoprotein cholesterol (LDL) levels in absolute values (**G**) as a percentage of change from pre-training values (**H**). ECC: eccentric cycling, CONC: concentric cycling.

No significant differences after ECC and CONC training in TAG levels (time effect, *p* = 0.4; training effect, *p* = 0.7; interaction effect, *p* > 0.9; Figure 6A), TC (time effect, *p* = 0.5; training effect, *p* = 0.6; interaction effect, *p* = 0.9; Figure 6C), HDL (time effect, *p* = 0.6; training effect, *p* = 0.6; interaction effect, *p* = 0.7; Figure 6E) and LDL (time effect, *p* = 0.6; training effect, *p* = 0.4; interaction effect, *p* = 0.7; Figure 6G) levels were observed. No differences were shown in percentages of changes from baseline in TAG, TC, and LDL between groups (Figure 6B,D,G), while HDL levels showed a tendency to increase after CONC compared to ECC (*p* = 0.06; Figure 6E).

## 4. Discussion

This study aimed to examine the effects of eccentric cycling (ECC) and conventional concentric cycling (CONC) training on plasma markers of oxidative stress, systemic inflammation, and cardiometabolic health in patients with moderate chronic obstructive pulmonary disease (COPD). The main findings of this study were: (1) maximal aerobic capacity and time-to-exhaustion (TTE) performance were maintained after 12 weeks of ECC and CONC training; (2) CONC induced an increase in total antioxidant capacity (TAC) and ECC induced a decrease in GPx activity at rest; (3) exercise-induced oxidative stress (i.e., TBARS) showed a smaller increase after CONC training; (4) insulin sensitivity and HDL were improved after CONC training only. Thus, our initial hypothesis was supported as CONC training induced greater improvements in oxidative stress at rest and after exercise, and cardiometabolic health markers changed more favorably after CONC training compared with ECC, while inflammatory markers remained unchanged after both training interventions.

We found that neither ECC nor CONC training improved maximal aerobic capacity (VO_2peak_) or time-to-exhaustion test (TTE) performance (Figure 1). COPD patients manifest pulmonary parenchyma fibrosis, airway remodeling, and dynamic hyperinflation, which may interfere with oxygen exchange [35]. Therefore, it is possible that no changes in oxygen consumption observed in these patients [20,36], were possibly due to the same reasons that the performance in the TTE test was also not changed after CONC or ECC. However, Porszasz et al. reported that endurance training (35 min per session at 75% of the peak work rate attained on incremental test) induced a 20% increase in PO_max_, which was accompanied by an increase in TTE performance [37]. These equivocal results in endurance performance may be due to the large heterogeneity in the primary factors (i.e., ventilatory response, dynamic hyperinflation) affecting exercise tolerance in COPD patients [38]. It is also possible that our training interventions were not sufficiently intense stimuli as to induce aerobic adaptations compared with previous study [37]. Mechanisms underpinning the absence of aerobic adaptations in this population may warrant further research.

Increased serum TBARS levels and reduced GPx activity have been associated with the disease severity of COPD patients [39]. We found that CONC training induced 137.9 ± 162.4% increase in TAC concentration (Figure 2A), while ECC training induced 17.2 ± 20.8% decrease in GPx activity (Figure 2C,D) measured at rest in moderate COPD patients. Equivocal results have been published previously. For instance, Rabinovich et al. showed that COPD patients decreased their antioxidant capacity after 8 weeks of concentric high-intensity interval training (HIIT), while healthy sedentary individuals increased their muscle antioxidant markers (i.e., glutathione levels; GSH). Interestingly, Zarrindast et al. showed that an 8-week moderate endurance training reduced lipid peroxidation (i.e., 8-iso PGF 2α), and increased TAC levels in elderly women [40]. It has been speculated that after regular training, there is an upregulation of the antioxidant enzymatic systems as an adaptive response. Specifically, superoxide dismutase (SOD), catalase (CAT), and glutathione peroxidase (GPx) activity act synergistically with non-enzymatic antioxidants to reduce the total antioxidant capacity. However, we did not observe changes in SOD and CAT concentrations in plasma, which is similar to Shin et al. which showed maintenance in TBARS levels and SOD activities at rest after 6 months of moderate endurance training, while GPx activity increased by 12% after training [41]. Interestingly, the same study reported that TBARS levels tended to increase (*p* = 0.052) in the control group after 6 months of endurance training in middle age obese women. Equivocal results regarding changes in oxidant and antioxidant agents in plasma after training interventions could be due to the heterogeneity in the disease severity of COPD patients. Overall, our results reveal that 12 weeks of moderate-intensity continuous endurance concentric training (CONC) may have positively changed the non-enzymatic antioxidant capacity and have maintained the TBARS levels of COPD patients at rest.

In our study, the TTE test was used to assess endurance capacity, and to induce a standardized (75% of individual PO_max_) physiological challenge to COPD patients to assess the response to exercise-induced oxidative stress before and after training interventions. Our results showed that the increase in TBARS levels following TTE was significantly smaller after CONC training (Figure 3E). Mercken et al. reported that COPD patients have elevated systemic and pulmonary oxidative stress at rest and in response to acute exercise compared with age-matched healthy control individuals [42]. This may suggest that healthy individuals can tolerate exercise-induced oxidative stress more effectively than COPD patients. Mercken et al. also showed for the first time that intensive supervised pulmonary rehabilitation (8-week training program) decreased exercise-induced oxidative stress following submaximal exercise [42]. This improved redox handling after exercise could be attributable to adaptive responses involving more efficient muscle oxidative metabolism (e.g., lesser mitochondrial ROS production) or a better capacity of endogenous antioxidant systems to handle oxidative stress. However, it is still not known if the systemic increase in oxidative stress markers in COPD patients is a reversible process and whether other exercise interventions could also modify this scenario as it has been shown to occur in other metabolic and cardiovascular diseases [43].

Systemic inflammatory markers were unchanged after ECC and CONC training (Figure 4). These results are similar to previous reports, in which circulating pro-inflammatory cytokines were unchanged in COPD patients regardless of endurance or strength-based training interventions [15,44]. Greater intensity and longer interventions may be necessary to modify inflammatory markers in this population.

We found a 30.8 ± 22.9% decrease in HOMA-IR (*p* = 0.0006) only after CONC training (Figure 5A,B). This result becomes relevant considering that our COPD patients had elevated baseline HOMA-IR index (ECC: 2.6 ± 1.2; CONC: 4.1 ± 2.3), suggesting insulin resistance [34]. Changes observed after CONC training were similar to the 20.4% decrease in HOMA-IR reported by Matos et al. after eight weeks of HIIT (at 80–110% of PO_max_) in obese patients with insulin resistance [45]. We have also previously shown similar decreases in HOMA-IR (–50%) after 12 weeks of concentric HIIT (at 70–100% maximum heart rate) in the same patients [46]. These positive changes induced by CONC training in the present study may be attributable to increases in skeletal muscle oxidative enzymes, mitochondrial biogenesis and glucose transporters induced by endurance exercise training [47]. The lack of changes after ECC training may be related to lesser metabolic demand imposed by eccentric contractions, which did not stimulate the skeletal muscle oxidative pathways and thus, no changes in oxidative capacity and cardiometabolic health markers were induced.

Although reductions in circulating lipids have been previously reported after eccentric training [31,48,49,50,51,52,53], we found no change in lipid profile markers after ECC training (Figure 6). An association has been observed between the magnitude of muscle damage induced by the eccentric contractions and changes in circulating lipids after eccentric training [54], so we speculate that the familiarization period and gradual increase in training workload in ECC may have avoided muscle damage. This could explain the lack of changes in lipid profile markers in the present study in comparison to previous eccentric training studies [31,48,49,50,51,52,53]. Interestingly, we found a tendency to increase HDL (*p* = 0.06) after CONC training (Figure 6F). For instance, Sillanpää et al. reported that endurance training (21 weeks) decreased total cholesterol (–5.6%), LDL (–5.4%), and TAG (–10.0%), without changes in HDL in healthy middle-aged men [55]. More recently, Boukabous et al. showed that endurance training (45 min per session at 55% heart rate reserve, 8 weeks) decreased LDL (–17.6%), and increased HDL (+7.6%) in older women [56]. Therefore, although both training programs (CONC and ECC) were performed at similar perceptual intensity (RPE of 11–13) and ECC performed ~3-fold greater workload than CONC, CONC showed larger improvements in markers of cardiometabolic health compared to ECC. However, lipid profile and insulin sensitivity changes reported in this study seem smaller compared to previous studies, which may be due to the lower training intensity (RPE = 13) of both interventions. Further studies should implement higher intensity and prolonged (more than 12 weeks) training interventions in order to induce greater cardiometabolic risk marker changes in COPD patients.

We acknowledge the limitations of our study. First, high variability in the outcome parameters may be due to differences in the severity of the disease of our patients; as we used patients with moderate COPD, this category may be too wide in symptoms and muscle dysfunction degrees, which increased variability. However, a stratified randomization was performed to distribute the participant in order to minimize this effect. Second, a small sample size was also recruited for this study, which also could have affected the variability of the results. Third, although all participants were diagnosed with moderate COPD, different clinical phenotypes exist [57]. It has been observed that emphysema and bronchiolitis can largely differ among patients of the same severity, which is evidenced by different levels of dyspnea while spirometric values are similar. The heterogeneity of the sample could have been reduced if more comprehensive inclusion criteria were used, e.g., exacerbation/hospitalizations per year. Finally, other factors such as daily physical activity and nutrition were not controlled and could have affected the results. Thus, our results need to be analyzed with caution as larger sample size clinical trials are needed to extrapolate these results to the general population.

## 5. Conclusions

In conclusion, moderate-intensity continuous concentric cycling training was more efficient to increase antioxidant capacity (enzymatic and non-enzymatic components) at rest and to improve exercise-induced oxidative stress control. Furthermore, it improved insulin sensitivity and HDL levels in moderate COPD patients to a greater extent than eccentric cycling training. Thus, moderate-intensity continuous concentric cycling training could be prescribed to reduce oxidative stress and improve markers of cardiometabolic health in comparison to eccentric cycling, which may be more indicated to increase muscle mass and functional performance [19]. Further research should focus on a combination of both concentric and eccentric cycling to target muscle dysfunction in COPD patients.

## Data Availability

Data are contained within the article.
